# Supplementary material for: Poly(ADP-ribosyl)ation associated changes in CTCF-chromatin binding and gene expression in breast cells
Source: Biochim Biophys Acta. 2018 Aug;1861(8):718–30. doi: 10.1016/j.bbagrm.2018.06.010 (PMC6074063; doi:10.1016/j.bbagrm.2018.06.010)
Supplement: Supplementary file 2 — Supplementary materials [file mmc2.pdf]

Supporting Materials

Poly(ADP-ribosyl)ation associated changes in CTCF-chromatin binding and gene expression in breast cells

Cell cycle arrest treatment with hydroxyurea and nocodazole

226LDM cells approximately 60-70% confluent were stripped off their spent medium and fresh culture medium containing 100 mM hydroxyurea was added to the flask. The treatment was administered as described by Docquier et al [1]. The cells were incubated for 24 hrs at 37°C and CO_2. After the end of the incubation time, the cells were incubated in fresh complete medium for 1hr at 37°C and $CO_{2.}$ After 1 hr the complete medium was aspirated off and fresh medium supplemented with 500 ng/ml nocodazole was added. After 24 hrs the detached cells were harvested and prepared for western blotting and chromatin immunoprecipitation. Quantification of the bends in western blots was performed using ImageJ [2] following the standard instructions provided by the developers.

Protein Immunoprecipitation

226LDM cells were trypsinised and then lysed by vortexing in BF2 (25 mM Tris/Hepes - pH 8.0, 2 mM EDTA, 0.5% Tween20, 0.5 M NaCl, 1in100 Halt Protease Inhibitors). The lysate was incubated on ice for 15 min and then equal volume of BF1 (25 mM Tris/Hepes - pH 8.0, 2 mM EDTA, 0.5% Tween20, 1in100 Halt Protease Inhibitors) was added. The cell lysate was pre-cleared by incubating 500 µl of the lysate in 50 µl of pre-blocked Protein A/Sepharose beads for 30 minutes at 4°C on a rotor shaker. The sample was then centrifuged at 200 g for 1 minute at RT and the pre-cleared supernatant was transferred into a fresh centrifuge tube. 50 µl of the sepharose beads were added to the pre-cleared lysate along with the antibody and the samples were incubated overnight at 4°C on a rotating wheel. On the following day, the immune-complexes were recovered by centrifugation at low speed for 1 min and the supernatant was removed. The pellet was washed three times with immunoprecipitation buffer (BF1+BF2) and each time the beads were collected with centrifugation at low speed for 1 minute. The sepharose was then lysed in SDS-lysis buffer and analysed by SDS-PAGE and western blot analysis.

Immunofluorescence (IF) staining on fixed cells

In immunofluorescence staining, which is based on the same principle as immunocytochemistry staining, an antibody is used to detect a specific protein. This antibody is appropriately tagged with a visible dye such as a fluorescent dye [3]. The IF staining was performed on adherent cells grown on cover slips. The cells were fixed with addition of 4% paraformaldehyde (PFA). After three washes with PBS/glycine (0.1 M), they were incubated for 15 min in boiling citrate buffer (10 mM citric acid, pH 6.0). After incubation for 20 min with permeabilization buffer (0.25% Triton / PBS) the cells were

washed thrice with 1 x PBS. The coverslips were placed in petri dishes, circled with hydrophobic marker on the slides and 90 μl of blocking buffer (0.05% Tween, 2% serum, 1% BSA / 1 x PBS) was added. A moist towel was put in the dish to keep the environment humid and the dish was covered and left on slow rocking in 4°C overnight. A 2 h long incubation with primary antibodies in buffer (0.05% Tween, 1% BSA, in 1 x PBS) was followed by three PBS washes. Incubation continued in the dark (covered with foil) with secondary antibodies conjugated with fluorescent dyes (e.g. FITC, TRITC) for 1hr. After 3 more washes with 1 x PBS, the coverslips were left to dry and then they were mounted to microscope slides with DAPI (4',6-diamidino-2-phenylindole, dilactate) mounting medium.

Microscopy

Immunofluorescence staining was observed under the Nikon Ti-Eclipse wide-field microscope which was used to capture the staining images. The visualisation tool used to view the images was Fusion FX viewer from Nikon.

Image quantification

Quantification of fluorescence intensity was performed on immunofluorescence staining images that were acquired with the same microscope settings using Fiji/ImageJ [2]. Briefly, the integrated pixel intensity (mean intensity per area) of the whole cell and the nucleus were measured for control and treated cells. The mean intensity of areas between cells was also measured to serve as background. For three different cells (n=3) from each condition, we calculated and compared the average mean intensity of control and treated cells in the whole cell and nucleus. After subtracting the background intensity in each case, the ratios of the resulting intensities were shown in the form of the box plot.

qPCR analysis

Real-time PCR was performed using ChIP samples to determine the disappearance of the peaks of chromatin density near CTCF binding sites illustrated in Supplementary Figure S9. The summits of the chromatin density peaks in control cells were determined from the green line Figure S9A for promoters of PARP3 and TP53. The following primer sequences were derived to detect the chromatin peaks for PARP3 and TP53:
PARP3 – FORWARD: TCAGAAGCGCCATGCTCA; REVERSE: AACAGCGGCTGCTCGTAAG;
TP53 – FORWARD: TATTCTCCGCCTGCATTTCT; REVERSE: TTCAAAGAAGGGGAGGGATT;
Each sample was amplified using SYBR green (SensiFAST SYBR no-ROX kit, Bioline) with the Lightcycler 96 instrument (ROCHE, CH). The manufacturer's instructions were followed regarding the reaction and cycling conditions. Briefly, 1 μl of ChIP DNA and 0.2 μM of primers were used to make up a 10 μl reaction. qPCR was performed separately for Input and for the no-antibody control. The no-antibody control did not produce qPCR signal and was assigned Ct value 40 as per standard instructions. qPCR intensity (fold-change) in the Input was compared between control and treated cells using the ΔΔCt method [4]. Following the ΔΔCt quantification, the chromatin density at the ChIP-seq peak summits near CTCF in control cells was assigned value one, and the density in treated cells was defined as a ratio to that in control cells.

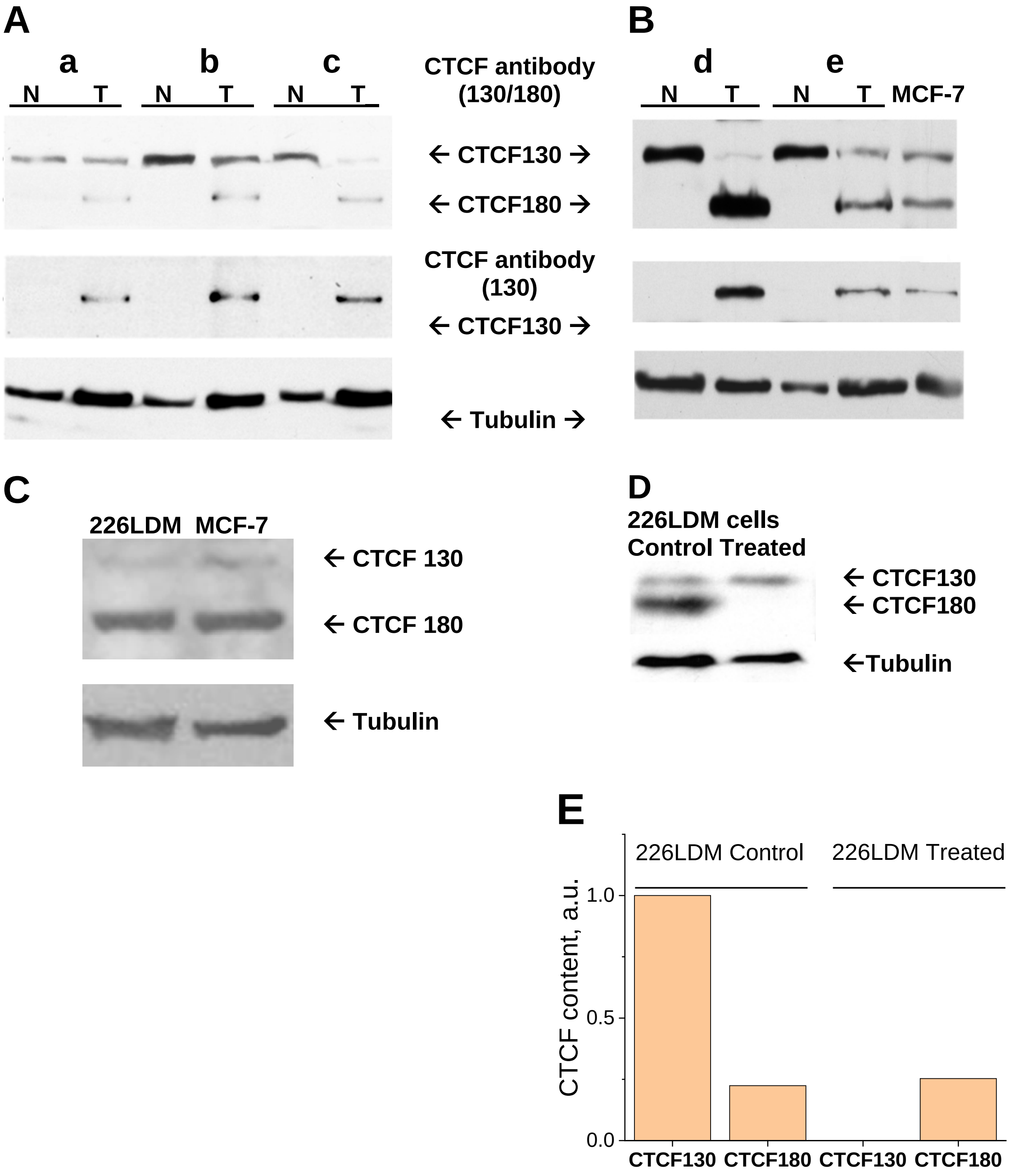

Supplementary Figure S1. Western Blot analyses of primary breast tissues and breast cell lines demonstrating the presence of two forms, CTF130 and CTCF180, with the antibodies pre-screened as previously described [1]**.**

A, B and C: CTCF migrates as 180 kDa protein in normal breast tissues, CTCF-130 appears in tumour breast tissues, and both forms of CTCF are present in MCF-7 breast cancer cells and 226LDM immortalized breast cells. **A.** Western blot analysis of three independent paired samples of normal and tumour tissues ("a", "b" and "c"). Tissue lysates (50 μg of the total protein) prepared as previously described [5] were resolved by SDS-PAGE, blotted and probed with the pre-screened anti-CTCF polyclonal antibody recognising CTCF180 and CTCF130 ("CTCF 130/180", upper panel), stripped and re-probed with the anti-CTCF antibody recognising only CTCF130 ("CTCF130", middle panel), re-stripped and re-probed with the anti-tubulin antibody ("Tubulin", lower panel). ***[Figure legend continues on the next page]***

[Figure S1 legend continues from the previous page]

B. Western blot analysis of two independent paired samples of normal (N) and tumour (T) tissues (“d” and “f”) together with the lysate from breast cancer cell line MCF7 (far right) performed as described above.

C. Western blot analysis of lysate from breast cancer cell line MCF7 and immortalized breast cells 226LDM probed with the pre-screened anti-CTCF polyclonal antibody recognising CTCF180 and CTCF130.

D. Western blot analysis of lysates from control and hydroxyurea/nocodazole treated 226LDM cells 226LDM cells were cell-cycle arrested by addition of 100mM hydroxyurea and 500ng/ml nocodazole in their culture medium (as described in the Supplementary materials and methods section) probed with the pre-screened anti-CTCF polyclonal antibody recognising CTCF180 and CTCF130. The development of the membranes was performed with the UptiLight™ chemiluminescence substrate. Tubulin was used as a loading control. Positions of CTCF-180, CTCF-130and tubulin are indicated. “N”- and “T” refer to normal and tumour breast tissues.

E. Quantification of the gel from panel D performed using ImageJ [2] following the standard instructions provided by the developers. In particular, the intensities of all bands were normalised by that of the corresponding tubulin band, and then normalised by the intensity of the band of CTCF130 in control. Following normalisation the value of CTCF130 in control was designated as 1.

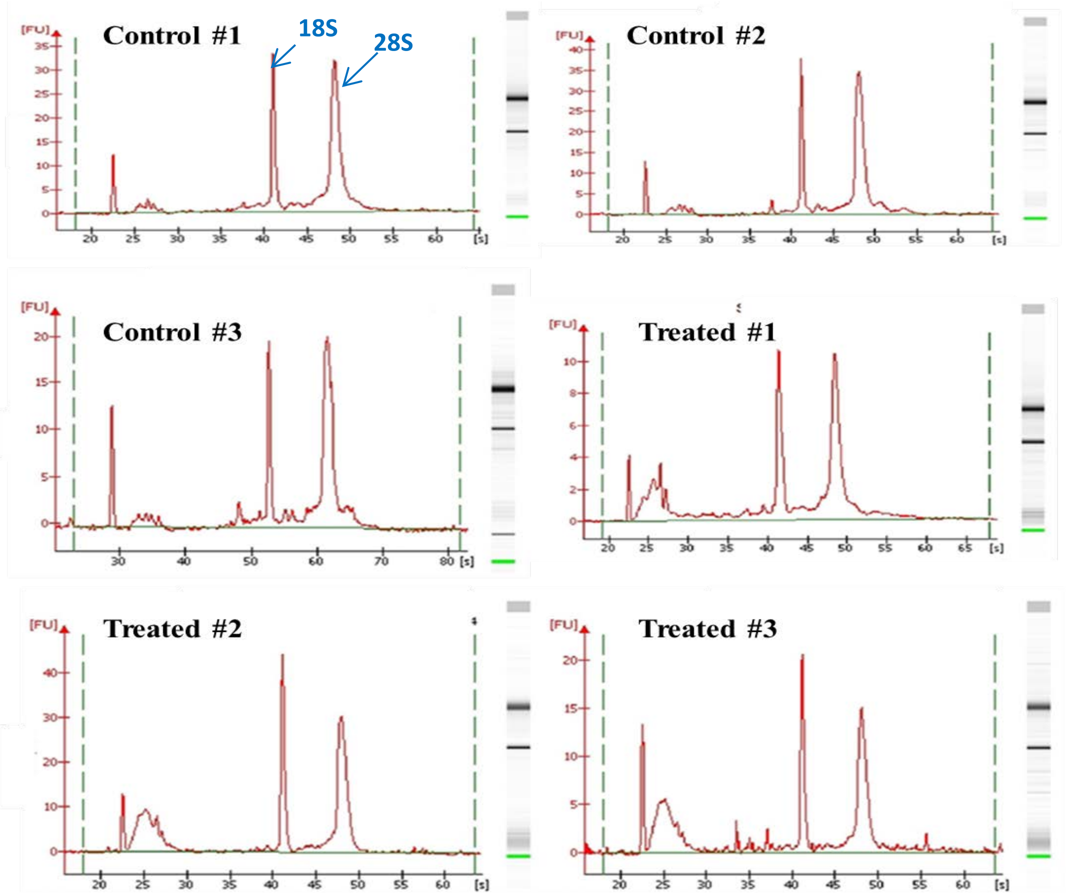

Supplementary Figure S2. Quality control of RNA isolated from control and treated 226LDM cells. Total RNA was isolated from control and treated 226LDM cells, each group in triplicates. To confirm the integrity of the content, the samples were run on a microfluidic chip using the Agilent Bioanalyzer 2100. The 28S and 18S ribosomal subunits are represented by peaks on the electropherograph and by bands on the gel placed on the right side of each graph.

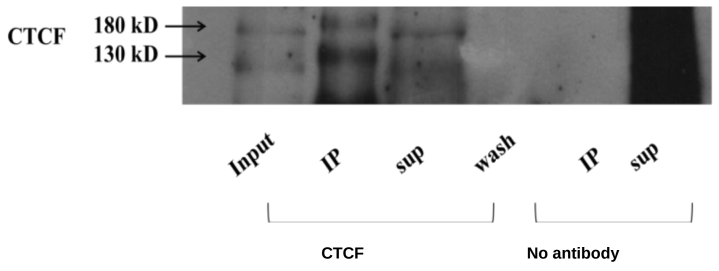

Supplementary Figure S3. Immunoprecipitation of CTCF in 226LDM cells with the anti-CTCF polyclonal antibody: Western blot analysis.

Protein extracts from untreated 226LDM cells were used in a series of protein immunoprecipitation experiments to confirm that both forms of CTCF (CTCF130 and CTCF180) can be immunoprecipitated with the selected anti-CTCF antibody (experimental details are described in the "Supplemental Materials and Methods" section). The proteins were resolved by SDS-PAGE, blotted, and probed with the selected anti-CTCF antibody. The visualization of the signal was performed using UptiLight™. Arrows indicate the positions of the two CTCF forms.

Keys:

Input: Pre-cleared and pre-blocked extracts (20 μl) from 226LDM cells used for the immunoprecipitation experiments.

IP: Immunoprecipitated proteins (5 μl) from lysed sepharose beads.

Sup: Supernatant material (20 μl) collected after centrifugation of beads with the immunoprecipitated proteins.

Wash: Material (20 μl) from the wash with the immunoprecipitation buffer (BF1+BF2).

No antibody: samples from the experiments performed using the same methods but without the selected CTCF antibody (used as control for the experiment.)

No antibody IP: Proteins (5 μl) from lysed sepharose beads.

Sup: Supernatant material (20 μl) collected after centrifugation of beads with the pre-cleared and pre-blocked extracts protein extracts.

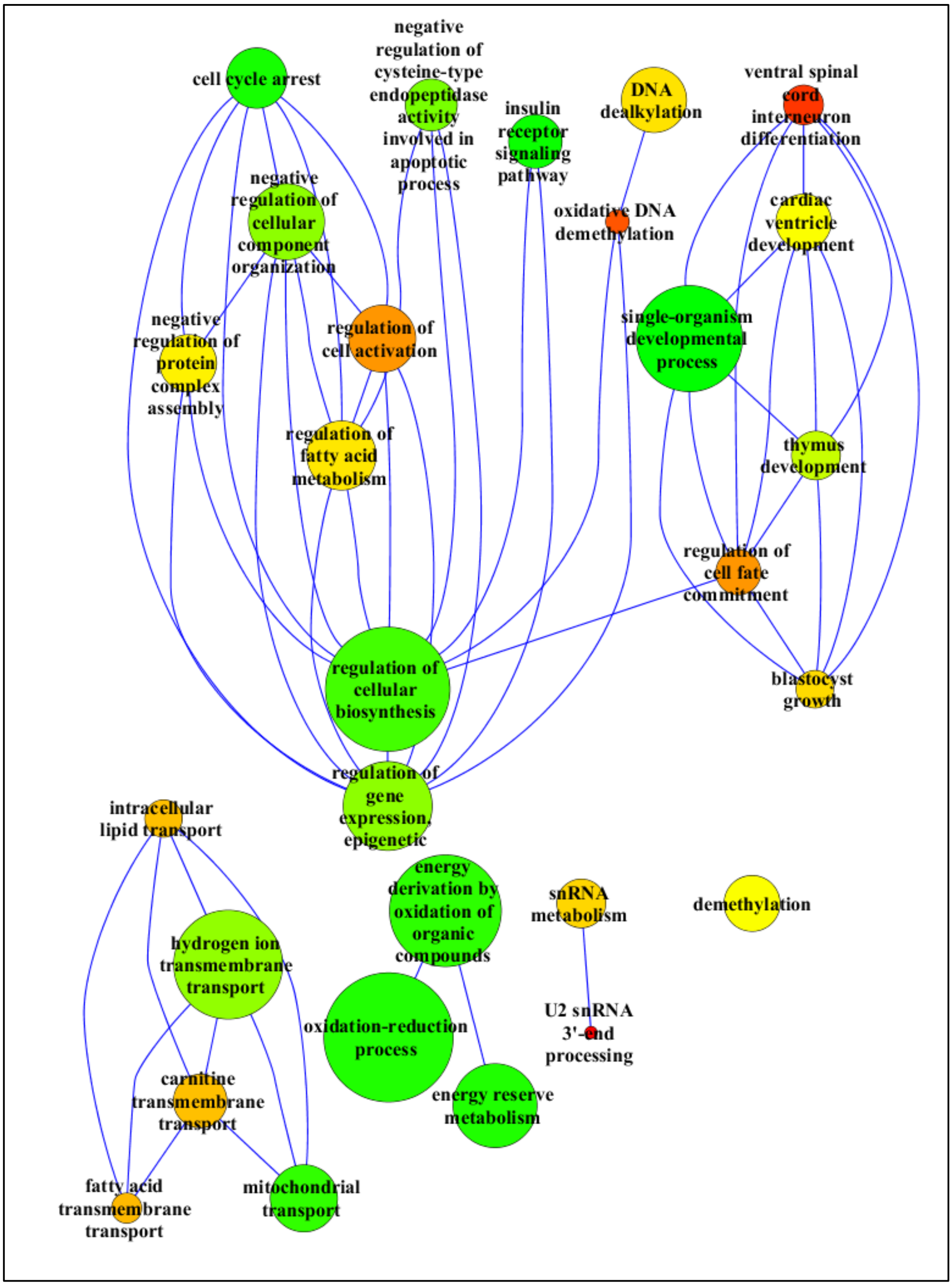

Supplementary Figure S4. Gene ontology analysis for the genes with up-regulated expression in cell-cycle blocked 226LDM cells treated with hydroxyurea and nocodazole.

Differential expression analysis was performed on control and cell-cycle blocked 226LDM cells with the DESEQ package. A gene ontology analysis was performed with the ensuing ranked list of significantly up-regulated genes. GO annotation terms were assigned to each gene and statistical Fanalysis and clustering of these terms was performed by the REViGO web server. The ontology relationships between the up-regulated genes are shown in the graph with nodes representing biological processes connected in a parent-child manner with edges. The size of the nodes represents the log-scales size of terms falling into the biological process described in the node label. The color of each node varies according to the p-value (from red to green, red representing the lowest p-value). The editing of the graph was performed with the CytoScape V3.2.1 software.

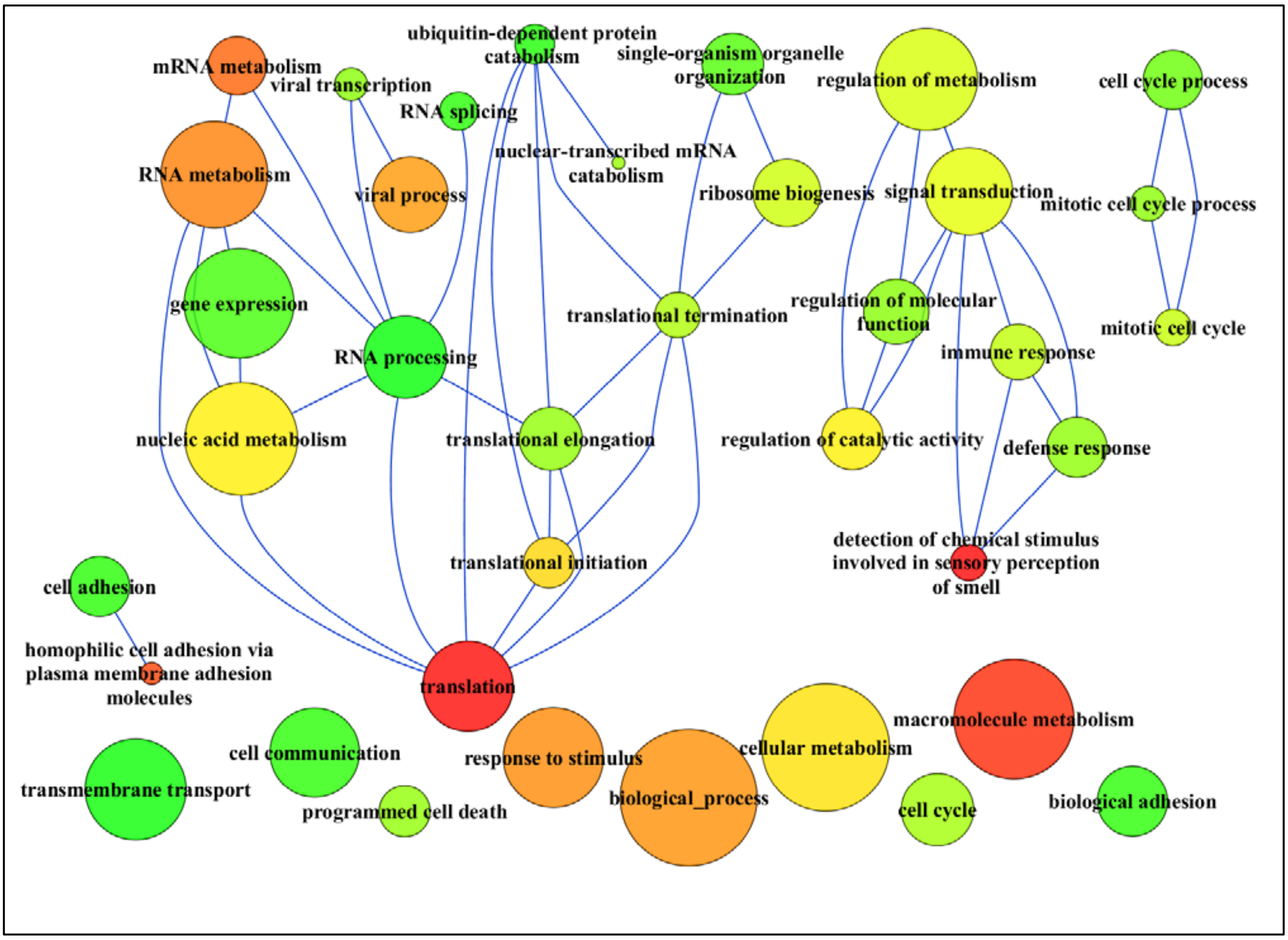

Supplementary Figure S5. Gene ontology analysis for the genes with down-regulated expression in cell-cycle arrested 226LDM cells treated with hydroxyurea and nocodazole.

Differential expression analysis was performed on control and cell-cycle blocked 226LDM cells with the DESEQ package. A gene ontology analysis was performed with the ensuing ranked list of significantly down-regulated genes. GO annotation terms were assigned to each gene and statistical analysis and clustering of these terms was performed by the REViGO web server. The ontology relationships between the down-regulated genes are shown in the graph with nodes representing biological processes connected in a parent-child manner with edges. The size of the nodes represents the log_size of terms falling into the biological process described in the node label. The color of each node varies according to the p-value (from red to green, red representing the lowest p-value). The editing of the graph was performed with the CytoScape V3.2.1 software.

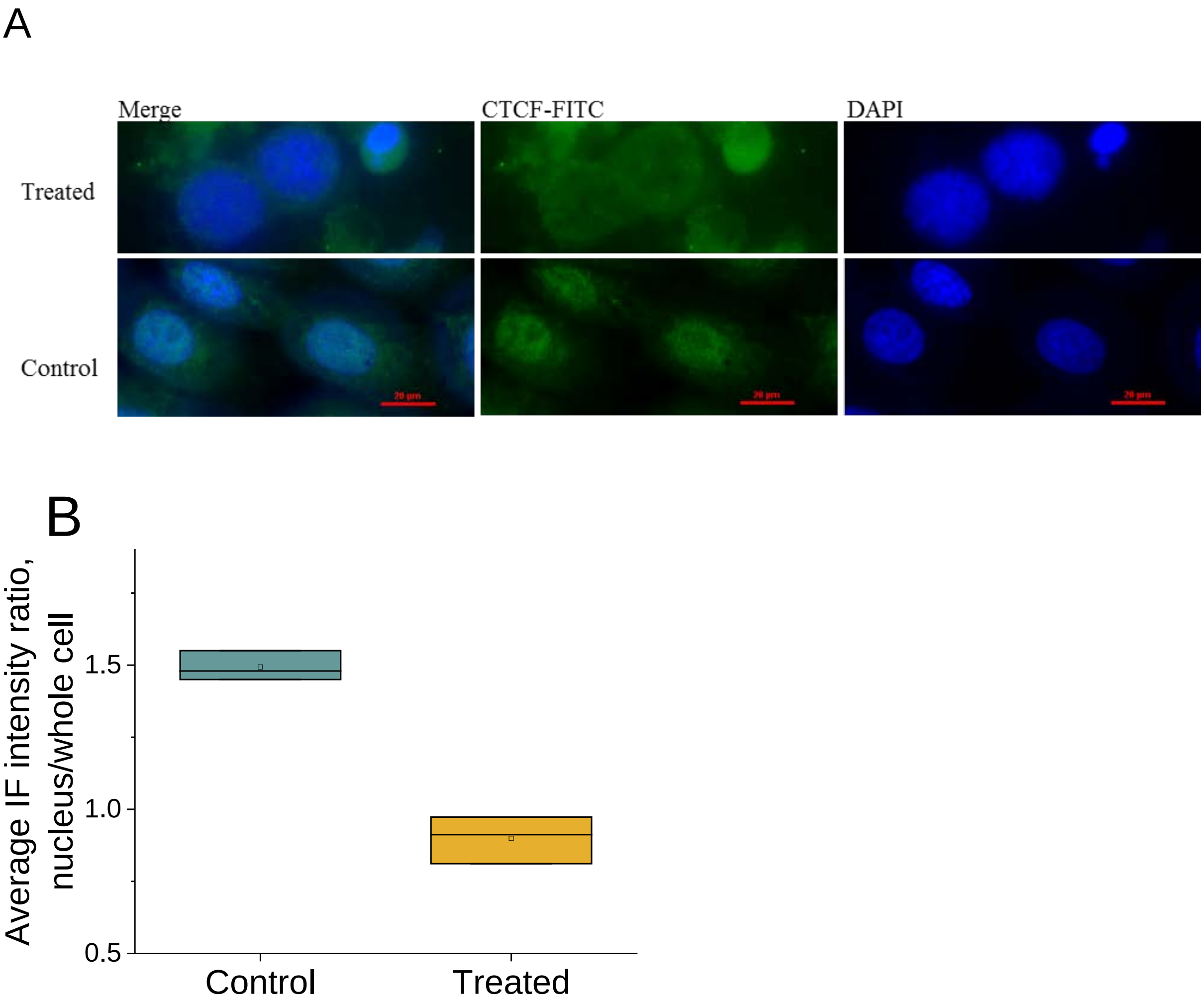

Supplementary Figure S6. Localization profile of CTCF in control and treated 226LDM cells.

A) CTCF immunofluorescence staining of control (untreated) and treated 226LDM cells using the polyclonal anti-CTCF antibody that recognizes both CTCF130 and CTCF180. In control cells the CTCF signal (green) appears *predominantly* diffused in the nuclear area. In treated cells staining is mostly detected in the cytoplasm. Blue colour shows DAPI staining of the nucleus.

B) Average CTCF immunofluorescence (IF) signal intensity ratio between the nucleus and the whole cell calculated separately for the treatment and control conditions. The quantification was performed using ImageJ [2] using three individual cells (N=3) on the same slide. All ratios of intensities were normalised by that in the control. The box height shows the standard deviation calculated based on three independent measurements (three cells). The horizontal line inside the box shows the median values.

Keys:

“CTCF-FITC” – Staining of CTCF and with secondary antibodies conjugated with a fluorescent dyes, (e.g. FITC.

“DAPI” – visualisation of nuclei with the DNA binding dye, DAPI (4’,6-diamidino-2-phenylindole, dilactate).

“Merge” – overlay of CTCF-FITC and DAPI fluorescent images.

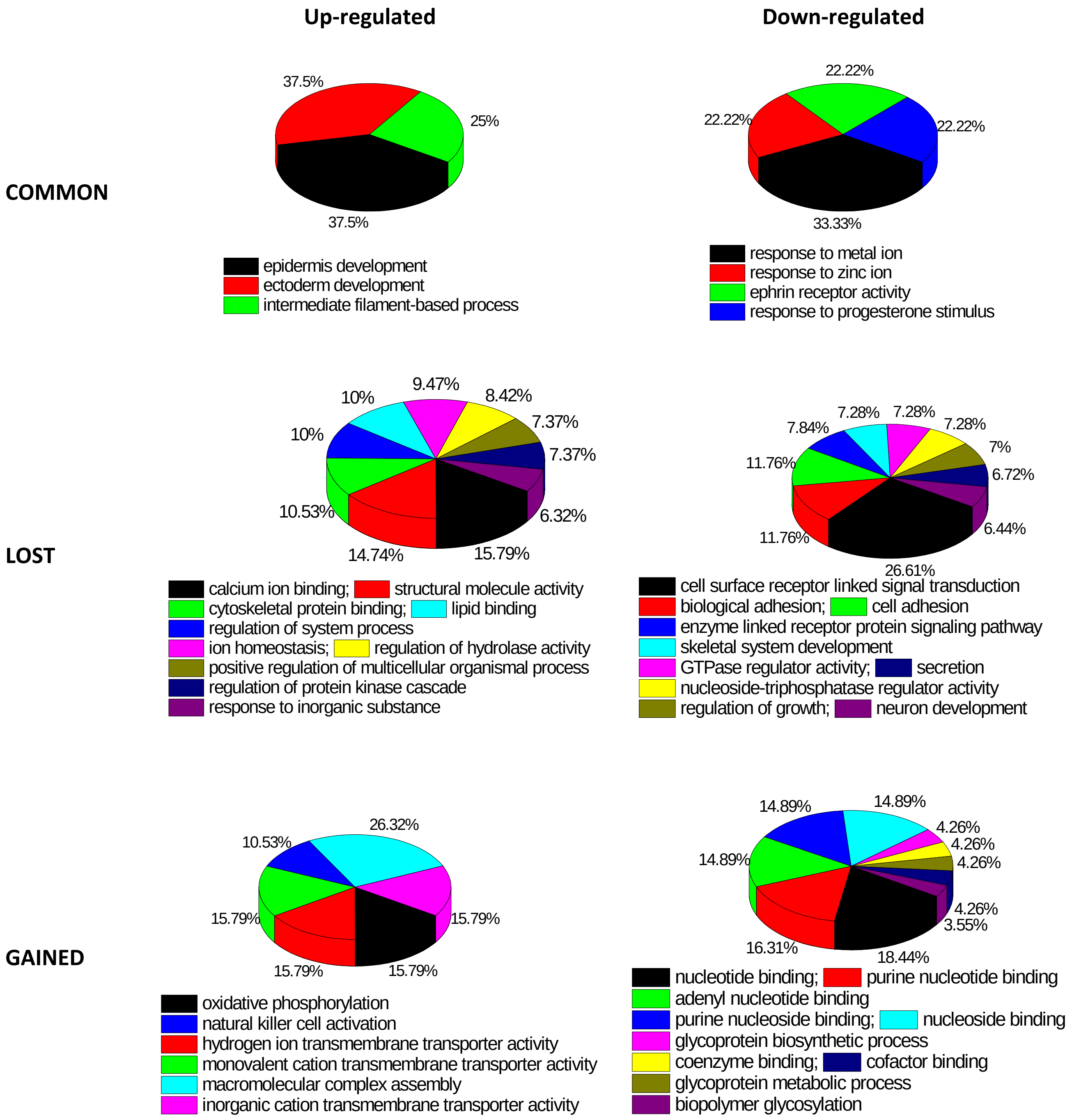

Supplementary Figure S7. Relative enrichment of gene ontology terms of the genes whose promoters contains CTCF sites within the interval [-10,000, +1000] around CTCF. The functional classification was performed using the same datasets as in Figure 1E.

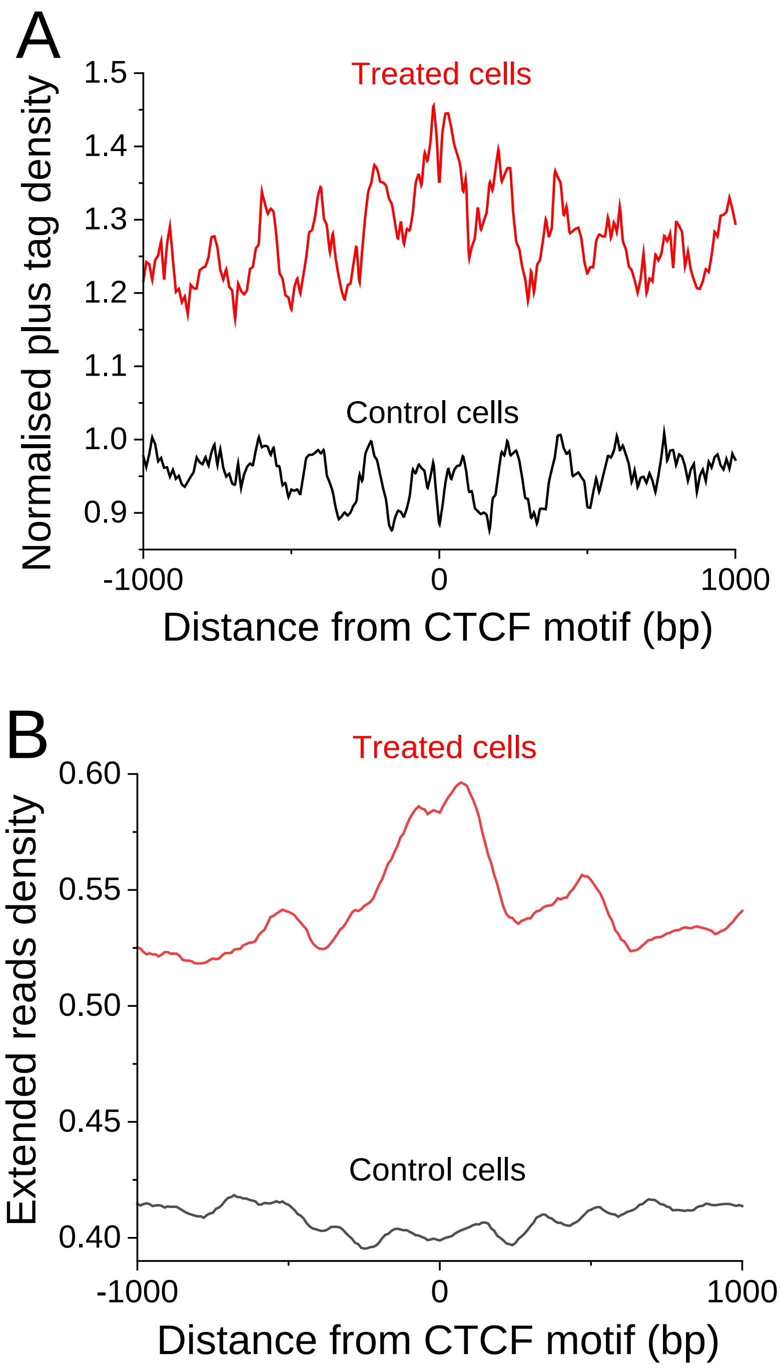

Supplementary Figure S8. Input tag read density changes reflect nucleosome repositioning near lost CTCF sites. (A) Average profile of tag density from Input (chromatin sonication without added antibody) around the centres of CTCF motifs inside lost CTCF peaks. Each peak corresponds to the preferred individual nucleosome location. The oscillation of peaks corresponds to the nucleosome repeat length. The profile obtained after the HOMER calculation was re-normalised by dividing each line by 0.002. (B) Aggregate nucleosome occupancy profile around the centres of CTCF binding motifs inside lost CTCF peaks, calculated based on the read density from panel (A). The calculation was performed using the default HOMER procedure, which includes extending the raw tags to achieve the average DNA fragment length estimated for a given sample. The original HOMER normalisation with respect to genome average is kept. In this normalisation values above 1 mean higher than genome-average, and below 1 mean lower than genome-average. Black lines – control (untreated) cells; red lines – treated cells.

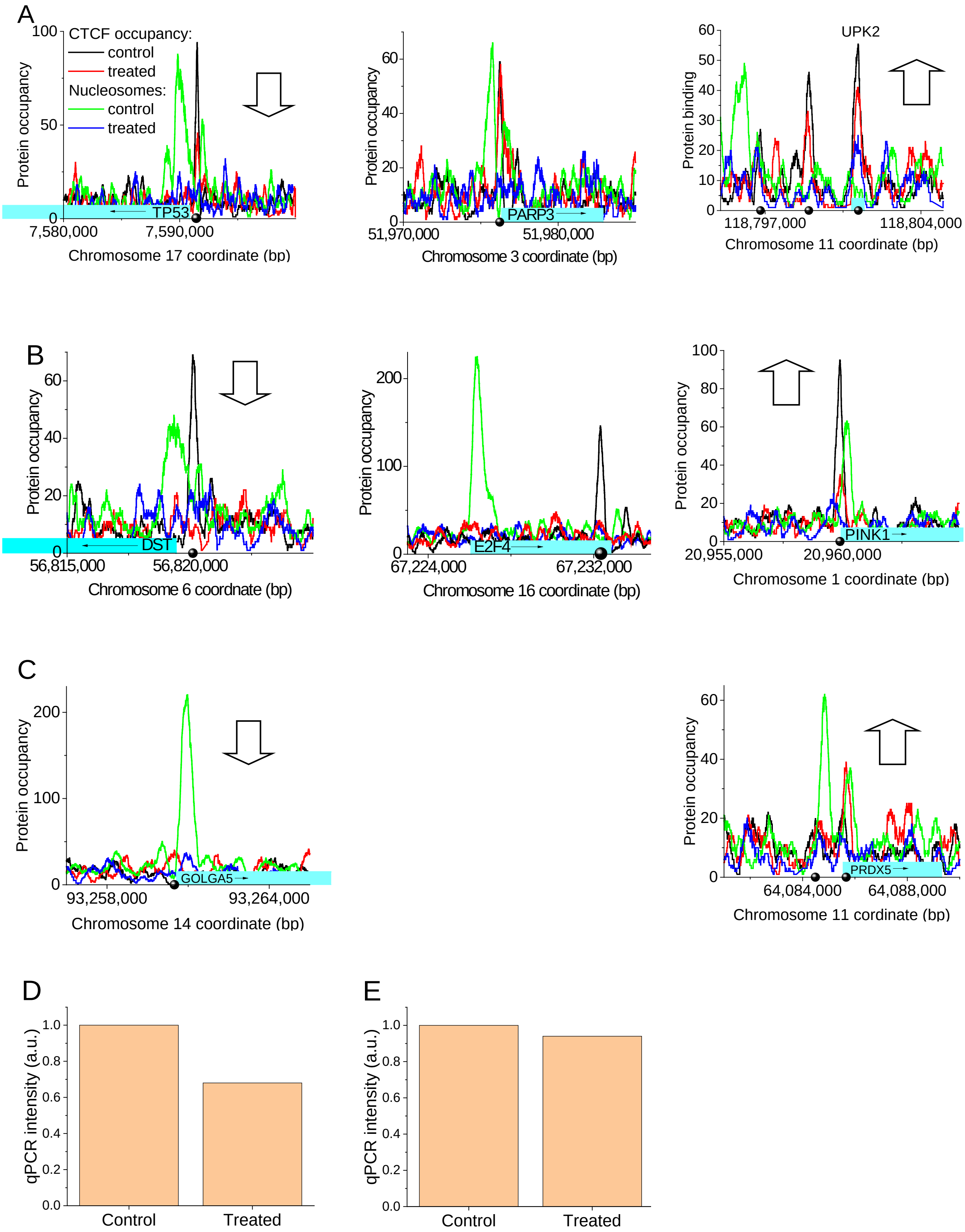

Supplementary Figure S9. Disappearance of the chromatin density peak near CTCF upon cell treatment. (A-C) Occupancy of CTCF and the corresponding ChIP-seq Input at exemplary promoters that contain common (A), lost (B) and gained (C) CTCF. (D-E) qPCR enrichment measurement at the summit of the chromatin peak positions given by the green line in panel A using Input and no-antibody control for promoters PARP3 (D) and TP53 (E). qPCR normalisation of Input samples was performed as described in Supplementary Methods.

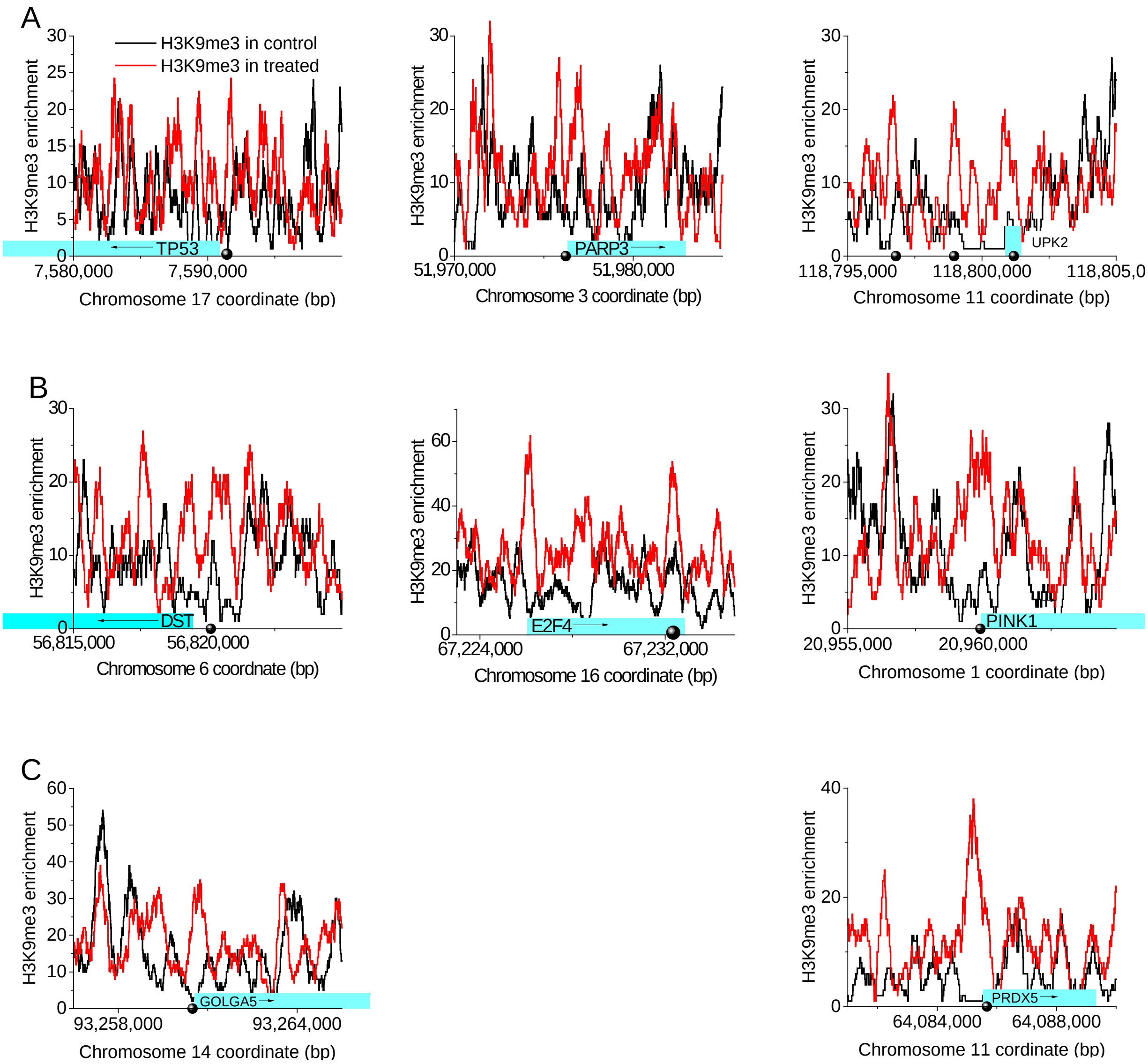

Supplementary Figure S10. Enrichment of H3K9me3 in control (black line) and treated cells (red line) at exemplary promoters that contain common (A), lost (B) and gained (C) CTCF. The same promoters as in Figure S9 are selected.

Supplemental References

[1] F. Docquier, G.X. Kita, D. Farrar, P. Jat, M. O'Hare, I. Chernukhin, S. Gretton, A. Mandal, L. Alldridge, E. Klenova, Decreased poly(ADP-ribosyl)ation of CTCF, a transcription factor, is associated with breast cancer phenotype and cell proliferation, Clinical cancer research : an official journal of the American Association for Cancer Research, 15 (2009) 5762-5771.
[2] C.T. Rueden, J. Schindelin, M.C. Hiner, B.E. DeZonia, A.E. Walter, E.T. Arena, K.W. Eliceiri, ImageJ2: ImageJ for the next generation of scientific image data, BMC Bioinformatics, 18 (2017) 529.
[3] J.A. Ramos-Vara, Technical aspects of immunohistochemistry, Vet Pathol, 42 (2005) 405-426.
[4] K.J. Livak, T.D. Schmittgen, Analysis of Relative Gene Expression Data Using Real-Time Quantitative PCR and the 2−ΔΔCT Method, Methods, 25 (2001) 402-408.
[5] V. D'Arcy, N. Pore, F. Docquier, Z.K. Abdullaev, I. Chernukhin, G.X. Kita, S. Rai, M. Smart, D. Farrar, S. Pack, V. Lobanenkov, E. Klenova, BORIS, a paralogue of the transcription factor, CTCF, is aberrantly expressed in breast tumours, British journal of cancer, 98 (2008) 571-579.
